# Understanding COVID-19 Vaccines Today: Are T-cells Key Players?

**DOI:** 10.3390/vaccines10060904

**Published:** 2022-06-06

**Authors:** Areez Shafqat, Tarek Z. Arabi, Belal N. Sabbah, Humzah S. Abdulkader, Shameel Shafqat, Adhil Razak, Junaid Kashir, Khaled Alkattan, Ahmed Yaqinuddin

**Affiliations:** 1 College of Medicine, Alfaisal University, Riyadh 11533, Saudi Arabia; tarabi@alfaisal.edu (T.Z.A.); bsabbah@alfaisal.edu (B.N.S.); habdulkader@alfaisal.edu (H.S.A.); arazak@alfaisal.edu (A.R.); jkashir@alfaisal.edu (J.K.); KKattan@alfaisal.edu (K.A.); ayaqinuddin@alfaisal.edu (A.Y.); 2 Medical College, Aga Khan University, P.O. Box 3500, Karachi, Pakistan; shameel.shafqat@scholar.aku.edu

**Keywords:** SARS-CoV-2, variants of concern, COVID-19 vaccines, T-cells, cell-mediated immunity

## Abstract

Severe acute respiratory syndrome coronavirus-2 (SARS-CoV-2) has heavily mutated since the beginning of the coronavirus-2019 (COVID-19) pandemic. In this regard, the so-called variants of concern (VOCs) feature mutations that confer increased transmissibility and evasion of antibody responses. The VOCs have caused significant spikes in COVID-19 cases, raising significant concerns about whether COVID-19 vaccines will protect against current and future variants. In this context, whereas the protection COVID-19 vaccines offer against the acquisition of infection appears compromised, the protection against severe COVID-19 is maintained. From an immunologic standpoint, this is likely underpinned by the maintenance of T-cell responses against VOCs. Therefore, the role of T-cells is essential to understanding the broader adaptive immune response to COVID-19, which has the potential to shape public policies on vaccine protocols and inform future vaccine design. In this review, we survey the literature on the immunology of T-cell responses upon SARS-CoV-2 vaccination with the current FDA-approved and Emergency Use Authorized COVID-19 vaccines.

## 1. Introduction

Since the beginning of the COVID-19 pandemic, a concerted effort from the scientific community has resulted in highly effective vaccines against the ancestral Wuhan strain. However, concerns subsequently arose about their efficacy against SARS-CoV-2 variants of concern (VOCs): VOCs acquired mutations in epitopes targeted by neutralizing antibodies (nAbs), resulting in the evasion of humoral responses. However, clinical trials evaluating COVID vaccines against the VOCs have yielded excellent protection from the severe disease. While humoral responses are essential in neutralizing viruses extracellularly, cellular responses comprising T-cells recognize and eliminate virus-infected cells. Therefore, although antibodies are crucial in preventing infection, cellular responses ameliorate disease severity, albeit this basic model does not consider other functions of antibodies, such as opsonization and antibody-mediated cellular cytotoxicity. Nevertheless, we utilize this model to explain the key findings of the literature surrounding the role of T-cell responses in underpinning the maintained efficacy of the currently approved COVID-19 vaccines.

## 2. Importance of T-cell Responses in Immunity to Human Coronaviruses (HCoVs)

SARS-CoV-2 is an enveloped single-stranded, positive-sense RNA virus with a large genome of approximately 30 kb, one of the largest among RNA viruses [1]. It belongs to the family of betacoronaviruses that includes the closely related SARS-CoV-1 and MERS-CoV-2 viruses that, although limited in incidence and prevalence globally, also cause severe respiratory infection in humans. On the other hand, the endemic common cold coronaviruses (CCCoVs) are highly prevalent—more than 90% of adults test positive for prior exposure, and seropositivity is near-ubiquitous in childhood—and include two betacoronaviruses, HKU-1 and OC43, and two alphacoronaviruses, 293E and NL63 [2,3].

Studying the humoral and cellular responses to other HCoVs provides an insight into the trajectory of long-term protective immunity against SARS-CoV-2. It also provides key data for characterizing the origin and nature of pre-existing cross-reactive immunity to SARS-CoV-2, which is detected in many COVID-19 and uninfected individuals (discussed below). The high rate of reinfection 12 months after initial CCoV infection suggests that sterilizing humoral immunity is absent, but the mild clinical symptoms of reinfection point to control by cellular responses that limit disease severity [4,5]. Similarly, cellular responses against SARS-CoV-1 remain robust even 17 years after the initial infection despite the waning of antibody responses [6]. Analyses of MERS-CoV have yielded similar results [7]; a recent study demonstrated that even seronegative individuals display cellular responses against MERS-CoV [8].

In summary, cell-mediated immunity to HCoVs, SARS-CoV-1, and MERS-CoV appears to be more robust and sustained than humoral responses. This is also seemingly the case for SARS-CoV-2: nAbs provide 87% protection against infection for 6 months [9], with sterilizing immunity remaining stable for 10 months, but waning afterward [10]. A study modeled the decay of nAb titers to reveal a significant decline over 250 days, which predisposes to reinfection [11]. Breakthrough infections in vaccinated individuals are also an issue. People double vaccinated with the BNT162b2 mRNA vaccine were seemingly protected from reinfection for 6 months, but serum nAbs then waned [12].

## 3. T-cell Contributions to Resolution of SARS-CoV-2 Infection and Memory

The limited understanding of the duration of infection and vaccine-induced protection against reinfection and breakthrough infection, respectively, has resulted in the implementation of vaccine booster doses at spaced intervals to enhance nAb titers. However, although conventional vaccines primarily aim to trigger humoral responses, the potential role of cellular immunity in the context of protection against COVID-19 disease progression should not be overlooked.

Though humoral responses are certainly important mediators in sterilizing immunity, their contribution to the resolution of SARS-CoV-2 infection is likely dispensable. For instance, patients with X-linked agammaglobulinemia and those receiving targeted anti-CD20 immunotherapy recover from COVID-19 without complications [13,14]. In contrast, robust CD4+ and CD8+ responses—the majority of which are directed against the S protein—are detected in convalescent COVID-19 patients [15,16] and are much more variable in acute severe COVID-19 [17]. Furthermore, CD4+ responses—not the antibody response—appear to be the best predictor of COVID-19 severity [17]. The early induction of T-cell responses is a major determinant of mild COVID-19, whereas delayed recruitment is associated with severe disease [18]. Lastly, asymptomatic individuals or those who recover from mild COVID-19 exhibit robust T-cell responses to SARS-CoV-2, despite remaining seronegative [16]. These findings show that while nAbs contributions pertain to the protection against SARS-CoV-2 infection, optimal T-cell responses ensure a favorable clinical outcome by clearing the infection.

Moreover, Dan et al. assessed immunologic memory 8 months post-SARS-CoV-2 infection [19]: nAb titers declined significantly over the 8 months, with approximately 25% of subjects becoming seronegative during this time. In contrast, cellular responses are more durable: 90% and 70% of individuals exhibited CD4+ and CD8+ responses, respectively, with most CD4+ cells adopting a follicular helper (T_FH_) phenotype. Furthermore, memory B-cells were detected in 100% of subjects after 8 months and even increased over time, being higher at 6 months than 1-month post-symptom onset [19]. Therefore, while nAb responses decay significantly over time, cellular and memory responses remain comparatively more stable.

Regarding vaccination, immunologic memory 6 months after the second dose of the mRNA-1273 Moderna vaccine features measurable CD4+ and CD8+ memory T-cell responses in 100% of individuals, with a significant portion of CD4+ memory assuming a T_FH_ phenotype [20]. An interesting comparative study evaluated the phenotypes of immunologic memory 6 months after vaccination by the BNT162b2 Pfizer, mRNA-1273 Moderna, Ad26.COV2.S Janssen, and NVX-CoV2373 Novavax vaccines. At 6 months after vaccination, although the mRNA vaccines featured significant reductions in antibody titers, T-cell and B-cell memory responses remained stable with 100% of individuals being positive for CD4+ memory T-cells [21].

Despite these encouraging results, these studies evaluated immunologic memory to SARS-CoV-2 in the blood. However, immunological memory is active at infection sites, draining lymph nodes (LNs), and secondary lymphoid organs. In this context, a recent study characterized tissue-level immunologic memory 6 months after SARS-CoV-2 infection to reveal abundant tissue-resident memory T and B-cells mainly in the lungs and lung-draining LNs with abundant germinal center reactions in the latter [22]. These results demonstrate that SARS-CoV-2 infection elicits a robust and coordinated cellular memory response [22].

Therefore, while cellular responses are essential to combating SARS-CoV-2 infection and ameliorating disease severity, they are also crucial in immunological memory against SARS-CoV-2. These immunologic responses attenuate clinical severity upon reinfection. Interestingly, recent studies have associated SARS-CoV-2 reactive memory T-cells with a reduced risk against reinfection, suggesting a potential role of T-cells in protecting against the acquisition of COVID-19 [23,24]. This could have major public health implications, particularly for vaccine boosting intervals, since only considering serology may underestimate infection- or vaccine-induced protective immunity. However, the lack of protection against reinfection/breakthrough infection with VOCs, despite the presence of robust cellular memory, argues against a role of T-cells in the protection against the acquisition of infection and is more consistent with a predominant role of nAbs in sterilizing immunity.

## 4. T-cells Cross-Recognize SARS-CoV-2 Variants including Omicron

The cellular entry of SARS-CoV-2 is mediated by its surface S protein, which binds to host angiotensin-converting enzyme-2 (ACE2) receptors. It is no surprise then, that nAbs, both natural and therapeutic, target regions of the S protein, particularly the receptor-binding domain (RBD) and N-terminal domain (NTD). Consequently, the S protein is subject to extreme selection pressures, resulting in mutations in the sequences targeted by nAbs. VOCs indeed harbor mutations in these sequences, resulting in antibody escape and reduced nAb activity against them [25,26] (Table 1). For example, the Omicron (B.1.1.529) variant features 32 new mutations relative to the ancestral Wuhan strain in the S protein concentrated in the RBD and NTD of S [27]. This clinically manifests in SARS-CoV-2, significantly evading antibody responses: the serum from convalescent patients shows a 44-fold lower efficacy in neutralizing Omicron than the Delta variant; the FDA-approved nAb cocktails of REGN-CoV-2 (casirivimab and imdevimab) and Eli-Lilly (etesevimab and bamlanivimab) fail to inhibit SARS-CoV-2 cellular entry; and the serum collected from vaccinees 10 months after the second BNT162b2 dose shows a 12-fold lower efficacy in neutralizing the Omicron variant compared to the Delta variant [28].

However, the current COVID-19 vaccines demonstrate >80–90% protection in preventing hospital admissions and emergency department visits after the completion of three doses [29] (Table 1). COVID-19 infection is indeed less severe among vaccinated patients than among those unvaccinated [29,30]. Therefore, although nAb responses appear significantly affected with increased chances of breakthrough infection, the vaccines remain effective in preventing severe disease. This is likely underpinned by durable cellular memory responses that VOCs cannot escape.

T-cells recognize and target a broader range of SARS-CoV-2 proteins compared to nAbs. Whereas nAb targets are limited to the viral surface, T-cells target surface as well as structural proteins. In this regard, though most studies show the S, M, and N proteins as being the immunodominant SARS-CoV-2 antigens targeted by T-cells, strong responses are also detected against ORF3, ORF8, ORF1ab, and nsp3, 4, 6, and 12 [31]. T-cells also recognize a broad range of epitopes within each antigen; over 1800 such epitopes have been identified so far [31,32]. Furthermore, Tarke et al. revealed that, on average, each individual recognizes 15–20 CD4+ and CD8+ epitopes each, suggesting that this may minimize the likelihood of VOCs escaping T-cell responses, since it is unlikely that VOCs acquire mutations in all these sequences [33]. In agreement with this, a recent study demonstrated CD4+ and CD8+ T-cells targeting 10–11 epitopes of SARS-CoV-2, of which 84% and 88%, respectively, of responses are preserved against Omicron [34]. Furthermore, 70–80% of T-cell responses against the Omicron S protein are preserved in both vaccinated and convalescent individuals [35]. Thus, the breadth of the T-cell repertoire generated against SARS-CoV-2 minimizes the likelihood of the evasion of T-cell responses by the VOCs.

**Table 1:** Efficacy of the COVID-19 vaccines in protecting against infection and severe disease caused by VOCs. Despite impaired protection against infection, protection against severe disease is maintained. This might be underpinned by preservation of T-cell responses against the VOCs.

Efficacy of the First-Generation Vaccines against VOCs.			
Vaccine	Strain	Protection against Infection	Protection against Severe Disease (%, 95% CI)
Pfizer-BioNTech (BNT162b2)	SARS-CoV-2 (Wuhan-Hu-1)	95% [36]	96.7% (73.9–99.9) [37]
	Delta (B.1.617.2)	Decreased neutralization titers by 2.2-fold compared to Wuhan-Hu-1 [38]	93% (84–96) [39]
	Omicron (B.1.1.529)	Decrease in neutralization of 44-fold compared to Wuhan-Hu-1 [40]	70% (62–76) [41]
Moderna (mRNA-1273)	SARS-CoV-2 (Wuhan-Hu-1)	93.2% (91.0–94.8) vs. symptomatic disease [42]	100% (CI could not be estimated) [43]
	Delta (B.1.617.2)	Only modestly decreased [44]	95.9%_unadj_ (86.9–98.7) [45]
	Omicron (B.1.1.529)	Decrease in neutralization of 33-fold compared to Wuhan-Hu-1 [40]	81.1%_unadj_ (29.8–94.9) [45]
AstraZeneca (ChAdOx1)	SARS-CoV-2 (Wuhan-Hu-1)	93% (at 83 IU/mL) [46]	89.9% (83.5–93.8) [47]
	Delta (B.1.617.2)	Eight-fold reduction in neutralizing titers compared to Wuhan-Hu-1 [48]	92% (75–97) [49]
	Omicron (B.1.1.529)	Decrease in neutralization of 36-fold compared to Wuhan-Hu-1 [40]	Approximately 56% [50]
J&J Janssen (Ad26.COV2.S)	SARS-CoV-2 (Wuhan-Hu-1)	100% [51]	57.7% (−2.6–82.5) [47]
	Delta (B.1.617.2)	1.6-fold reduction in neutralization sensitivity compared to Wuhan-Hu-1 [52]	81% (75–86) [53]
	Omicron (B.1.1.529)	Majority of samples did not reveal neutralizing activity [27]	74% (57–84) [41]

## 5. Pre-Existing SARS-CoV-2 T-cell Cross-Reactivity

Numerous reports early in the pandemic demonstrated the presence of SARS-CoV-2 reactive T-cells in healthy donor blood samples, some of which were collected even before the pandemic [15,54,55,56,57]. Based on subsequent studies, these T-cells were shown to likely derive from exposure to CCCoVs, although other potential origins have been reported, including prior exposure to influenza or CMV [58,59,60,61].

Studies then investigated the impact of pre-existing T-cell reactivity on the clinical outcome of COVID-19 and on immune responses post-vaccination. In this regard, pre-existing T-cell immunity to SARS-CoV-2 is associated with a less severe COVID-19 clinical course [62,63]. To explain these findings immunologically, recall that the timeliness of the T-cell response is a key determinant of COVID-19 severity. Therefore, the recruitment of pre-existing T-cell memory responses upon infection may confer a kinetic advantage in favor of SARS-CoV-2 clearance [32]. In this regard, Loyal et al. demonstrated the recruitment of cross-reactive T-cells after SARS-CoV-2 infection and after vaccination with the BNT162b2 mRNA vaccine, resulting in more robust cellular and humoral responses in both instances [64]. Mateus and colleagues reported similar findings after a low-dose mRNA-1273 Moderna vaccination, whereby pre-existing T-cells enhanced spike-specific T_FH_ and antibody responses [20].

Therefore, pre-existing T-cell memory against SARS-CoV-2 originates from prior exposure to the CCCoVs and augments the adaptive response against SARS-CoV-2, both after infection and vaccination. Lastly, two studies uncovered a protective function of pre-existing memory T-cells against SARS-CoV-2 infection [23,24]. However, comparing the T-cell repertoire induced by SARS-CoV-2 infection with that of pre-existing T-cells has revealed 227 new epitopes targeted by infection-induced T-cell responses, suggesting a limited function of pre-existing immunity to sterilizing immunity, since infection elicits largely a new epitope repertoire [33].

## 6. Discussion

In this review, we covered the major findings related to the immunology of T-cells contributing to maintained vaccine efficacy. T-cells are crucial in clearing SARS-CoV-2 infection—even without seroconversion—manifested clinically as a more favorable COVID-19 prognosis. T-cells also remain remarkably more stable after SARS-CoV-2 infection and vaccination compared to nAbs, which wane significantly after 6 months. The broad range of epitopes targeted by T-cells allows for largely preserved responses to SARS-CoV-2 VOCs. Lastly, pre-existing T-cell memory responses appear to have a beneficial effect on the robustness of the immune response after infection and vaccination. However, numerous uncertainties still exist regarding cellular immunity to SARS-CoV-2 that must be addressed by future research.

Future SARS-CoV-2 variants will inevitably emerge, which may have accumulated more spike mutations than Omicron and evade humoral responses to an even greater extent. Future clinical trials that will assess vaccine efficacy necessarily have to measure T-cell responses, and not solely focus on nAbs, since the latter can potentially be severely compromised, leading to significant public concern and reservations over the current vaccines [65].

Since pre-existing T-cell immunity may be associated with less severe COVID-19, better characterization of the T-cell response against other HCoVs may identify suitable candidate antigens to include in future vaccines. If the T-cell response against these sequences is conserved in SARS-CoV-2, these vaccines will broaden the vaccine-induced T-cell repertoire against SARS-CoV-2 and protect from other zoonotic coronaviruses with pandemic-causing potential [32]. Alternatively, elucidating protective CD4+ and CD8+ phenotypes against disease progression may allow for the inclusion of specific SARS-CoV-2 sequences into second-generation vaccines to broaden the vaccine-induced T-cell repertoire [66].

However, population-based data of T-cell responses is currently lacking and are needed to identify correlates of protection that can be leveraged to protect against severe disease via vaccination and identify at-risk individuals who may benefit from booster doses over others, which will help inform future public health strategies [65]. Furthermore, direct clinical evidence associating specific T-cell repertoires and COVID-19 severity is currently lacking and defining what constitutes a protective T-cell response upon vaccination or infection needs to be the focus of future studies. In this regard, the relative contributions of nAbs and T-cells to the protection against infection and disease progression need to be precisely delineated. However, a major hindrance in conducting such a study is isolating nAb and T-cell responses. For example, good nAbs may protect against infection and control disease progression, but this would make the precise role of T-cells challenging to disentangle [67]. In addition, such a study requires a considerable sample size due to the low incidence of severe disease, especially in vaccinated individuals. A recent review to address this dilemma advocated focusing on breakthrough infections and then assessing nAb and T-cell responses as independent and combined predictors of clinical outcome and/or viral decay [67]. Undoubtedly, better characterizing the adaptive response against SARS-CoV-2, with a focus on cellular immunity, will inform future diagnostic approaches, vaccine designs, and public health strategies.

A major step to delineating protective versus maladaptive T-cell responses would be standardizing the laboratory approaches to assess them. An excellent review by Vardhana et al. surveyed the various laboratory approaches to evaluate T-cell responses, many of which have the potential to be applied at a population level [65]. Combining T-cell responses, and serology in parallel, in clinical studies will allow us to holistically appreciate the adaptive response to SARS-CoV-2 infection and vaccination. Furthermore, this will provide a more accurate estimation of the prevalence of COVID-19, since many asymptomatic or mildly affected individuals are seronegative but do develop robust T-cell responses. Lastly, we recommend standardizing the approach to measuring T-cell responses, since this will also allow for more accurate comparisons between studies, which is a current limitation.

## References

[1] Cao C., Cai Z., Xiao X., Rao J., Chen J., Hu N., Yang M., Xing X., Wang Y., Li M. (2021). The architecture of the SARS-CoV-2 RNA genome inside virion. Nat. Commun..

[2] Gorse G.J., Patel G.B., Vitale J.N., O’Connor T.Z. (2010). Prevalence of antibodies to four human coronaviruses is lower in nasal secretions than in serum. Clin. Vaccine Immunol..

[3] Moss P. (2022). The T cell immune response against SARS-CoV-2. Nat. Immunol..

[4] Edridge A.W.D., Kaczorowska J., Hoste A.C.R., Bakker M., Klein M., Loens K., Jebbink M.F., Matser A., Kinsella C.M., Rueda P. (2020). Seasonal coronavirus protective immunity is short-lasting. Nat. Med..

[5] Tan H.-X., Lee W.S., Wragg K.M., Nelson C., Esterbauer R., Kelly H.G., Amarasena T., Jones R., Starkey G., Wang B.Z. (2021). Adaptive immunity to human coronaviruses is widespread but low in magnitude. Clin. Transl. Immunol..

[6] Le Bert N., Tan A.T., Kunasegaran K., Tham C.Y.L., Hafezi M., Chia A., Chng M.H.Y., Lin M., Tan N., Linster M. (2020). SARS-CoV-2-specific T cell immunity in cases of COVID-19 and SARS, and uninfected controls. Nature.

[7] Zhao J., Abeer N.A., Salim A.B., Waleed A.A., Ahmad A.B., Atef M.N., Laila A.L., Mohammed G.A., Al Gethamy Manal M., Ashraf M.D. (2017). Recovery from the Middle East respiratory syndrome is associated with antibody and T cell responses. Sci. Immunol..

[8] Mok C.K.P., Zhu A., Zhao J., Lau E.H.Y., Wang J., Chen Z., Zhuang Z., Wang Y., Alshukairi A.N., Baharoon S.A. (2021). T-cell responses to MERS coronavirus infection in people with occupational exposure to dromedary camels in Nigeria: An observational cohort study. Lancet Infect. Dis..

[9] Hall V.J., Foulkes S., Charlett A., Atti A., Monk E.J.M., Simmons R., Wellington E., Cole M.J., Saei A., Oguti B. (2021). SARS-CoV-2 infection rates of antibody-positive compared with antibody-negative health-care workers in England: A large, multicentre, prospective cohort study (SIREN). Lancet.

[10] Murchu E.O., Byrne P., Carty P.G., De Gascun C., Keogan M., O’Neill M., Harrington P., Ryan M. (2022). Quantifying the risk of SARS-CoV-2 reinfection over time. Rev. Med. Virol..

[11] Khoury D.S., Cromer D., Reynaldi A., Schlub T.E., Wheatley A.K., Juno J.A., Subbarao K., Kent S.J., Triccas J.A., Davenport M.P. (2021). Neutralizing antibody levels are highly predictive of immune protection from symptomatic SARS-CoV-2 infection. Nat. Med..

[12] Hall V., Foulkes S., Insalata F., Kirwan P., Saei A., Atti A., Wellington E., Khawam J., Munro K., Cole M. (2022). Protection against SARS-CoV-2 after Covid-19 Vaccination and Previous Infection. N. Engl. J. Med..

[13] Soresina A., Moratto D., Chiarini M., Paolillo C., Baresi G., Focà E., Bezzi M., Baronio B., Giacomelli M., Badolato R. (2020). Two X-linked agammaglobulinemia patients develop pneumonia as COVID-19 manifestation but recover. Pediatr. Allergy Immunol..

[14] Montero-Escribano P., Matías-Guiu J., Gómez-Iglesias P., Porta-Etessam J., Pytel V., Matias-Guiu J.A. (2020). Anti-CD20 and COVID-19 in multiple sclerosis and related disorders: A case series of 60 patients from Madrid, Spain. Mult. Scler. Relat. Disord..

[15] Grifoni A., Weiskopf D., Ramirez S.I., Mateus J., Dan J.M., Moderbacher C.R., Rawlings S.A., Sutherland A., Premkumar L., Jadi R.S. (2020). Targets of T Cell Responses to SARS-CoV-2 Coronavirus in Humans with COVID-19 Disease and Unexposed Individuals. Cell.

[16] Sekine T., Perez-Potti A., Rivera-Ballesteros O., Strålin K., Gorin J.-B., Olsson A., Llewellyn-Lacey S., Kamal H., Bogdanovic G., Muschiol S. (2020). Robust T Cell Immunity in Convalescent Individuals with Asymptomatic or Mild COVID-19. Cell.

[17] Moderbacher C.R., Ramirez S.I., Dan J.M., Grifoni A., Hastie K.M., Weiskopf D., Belanger S., Abbott R.K., Kim C., Choi J. (2020). Antigen-Specific Adaptive Immunity to SARS-CoV-2 in Acute COVID-19 and Associations with Age and Disease Severity. Cell.

[18] Sette A., Crotty S. (2021). Adaptive immunity to SARS-CoV-2 and COVID-19. Cell.

[19] Dan Jennifer M., Mateus J., Kato Y., Kathryn M.H., Esther D.Y., Caterina E.F., Grifoni A., Sydney I.R., Haupt S., Frazier A. (2021). Immunological memory to SARS-CoV-2 assessed for up to 8 months after infection. Science.

[20] Mateus J., Dan Jennifer M., Zhang Z., Moderbacher C.R., Lammers M., Goodwin B., Sette A., Crotty S., Weiskopf D. (2021). Low-dose mRNA-1273 COVID-19 vaccine generates durable memory enhanced by cross-reactive T cells. Science.

[21] Zhang Z., Mateus J., Coelho C.H., Dan J.M., Moderbacher C.R., Gálvez R.I., Cortes F.H., Grifoni A., Tarke A., Chang J. (2022). Humoral and cellular immune memory to four COVID-19 vaccines. bioRxiv.

[22] Maya M.L.P., Rybkina K., Kato Y., Kubota M., Matsumoto R., Nathaniel I.B., Zhang Z., Kathryn M.H., Grifoni A., Weiskopf D. (2021). SARS-CoV-2 infection generates tissue-localized immunological memory in humans. Sci. Immunol..

[23] Wyllie D., Jones H.E., Mulchandani R., Trickey A., Taylor-Phillips S., Brooks T., Charlett A., Ades A.E., Moore P., EDSAB-HOME investigators (2021). SARS-CoV-2 responsive T cell numbers and anti-Spike IgG levels are both associated with protection from COVID-19: A prospective cohort study in keyworkers. medRxiv.

[24] Kundu R., Narean J.S., Wang L., Fenn J., Pillay T., Fernandez N.D., Conibear E., Koycheva A., Davies M., Tolosa-Wright M. (2022). Cross-reactive memory T cells associate with protection against SARS-CoV-2 infection in COVID-19 contacts. Nat. Commun..

[25] Yaqinuddin A., Shafqat A., Kashir J., Alkattan K. (2021). Effect of SARS-CoV-2 Mutations on the Efficacy of Antibody Therapy and Response to Vaccines. Vaccines.

[26] Hurt A.C., Wheatley A.K. (2021). Neutralizing Antibody Therapeutics for COVID-19. Viruses.

[27] Schmidt F., Muecksch F., Weisblum Y., Da Silva J., Bednarski E., Cho A., Wang Z., Gaebler C., Caskey M., Nussenzweig M.C. (2021). Plasma Neutralization of the SARS-CoV-2 Omicron Variant. N. Engl. J. Med..

[28] Hoffmann M., Krüger N., Schulz S., Cossmann A., Rocha C., Kempf A., Nehlmeier I., Graichen L., Moldenhauer A.-S., Winkler M.S. (2022). The Omicron variant is highly resistant against antibody-mediated neutralization: Implications for control of the COVID-19 pandemic. Cell.

[29] Lauring A.S., Tenforde M.W., Chappell J.D., Gaglani M., Ginde A.A., McNeal T., Ghamande S., Douin D.J., Talbot H.K., Casey J.D. (2022). Clinical severity of, and effectiveness of mRNA vaccines against, covid-19 from omicron, delta, and alpha SARS-CoV-2 variants in the United States: Prospective observational study. BMJ.

[30] Ferdinands J.M., Rao S., Dixon B.E., Mitchell P.K., DeSilva M.B., Irving S.A., Lewis N., Natarajan K., Stenehjem E., Grannis S.J. (2022). Waning 2-Dose and 3-Dose Effectiveness of mRNA Vaccines Against COVID-19-Associated Emergency Department and Urgent Care Encounters and Hospitalizations Among Adults During Periods of Delta and Omicron Variant Predominance —VISION Network, 10 States, August 2021–January 2022. MMWR Morb. Mortal. Wkly. Rep..

[31] Grifoni A., Sidney J., Vita R., Peters B., Crotty S., Weiskopf D., Sette A. (2021). SARS-CoV-2 human T cell epitopes: Adaptive immune response against COVID-19. Cell Host Microbe.

[32] Dan J., da Silva Antunes R., Grifoni A., Weiskopf D., Crotty S., Sette A. (2022). Observations and perspectives on adaptive immunity to SARS-CoV-2. Clin. Infect. Dis..

[33] Tarke A., Sidney J., Kidd C.K., Dan J.M., Ramirez S.I., Yu E.D., Mateus J., da Silva Antunes R., Moore E., Rubiro P. (2021). Comprehensive analysis of T cell immunodominance and immunoprevalence of SARS-CoV-2 epitopes in COVID-19 cases. Cell Rep. Med..

[34] Tarke A., Coelho C.H., Zhang Z., Dan J.M., Yu E.D., Methot N., Bloom N.I., Goodwin B., Phillips E., Mallal S. (2022). SARS-CoV-2 vaccination induces immunological T cell memory able to cross-recognize variants from Alpha to Omicron. Cell.

[35] Keeton R., Tincho M.B., Ngomti A., Baguma R., Benede N., Suzuki A., Khan K., Cele S., Bernstein M., Karim F. (2022). T cell responses to SARS-CoV-2 spike cross-recognize Omicron. Nature.

[36] Polack F.P., Thomas S.J., Kitchin N., Absalon J., Gurtman A., Lockhart S., Perez J.L., Marc G.P., Moreira E.D., Zerbini C. (2020). Safety and Efficacy of the BNT162b2 mRNA Covid-19 Vaccine. N. Engl. J. Med..

[37] Thomas S.J., Moreira E.D., Kitchin N., Absalon J., Gurtman A., Lockhart S., Perez J.L., Marc G.P., Polack F.P., Zerbini C. (2021). Safety and Efficacy of the BNT162b2 mRNA Covid-19 Vaccine through 6 Months. N. Engl. J. Med..

[38] Muik A., Bonny G.L., Wallisch A.-K., Bacher M., Mühl J., Reinholz J., Ozhelvaci O., Beckmann N., de la Caridad Güimil Garcia R., Poran A. (2022). Neutralization of SARS-CoV-2 Omicron by BNT162b2 mRNA vaccine–elicited human sera. Science.

[39] Tartof S.Y., Slezak J.M., Fischer H., Hong V., Ackerson B.K., Ranasinghe O.N., Frankland T.B., Ogun O.A., Zamparo J.M., Gray S. (2021). Effectiveness of mRNA BNT162b2 COVID-19 vaccine up to 6 months in a large integrated health system in the USA: A retrospective cohort study. Lancet.

[40] Cameroni E., Bowen J.E., Rosen L.E., Saliba C., Zepeda S.K., Culap K., Pinto D., VanBlargan L.A., De Marco A., di Iulio J. (2022). Broadly neutralizing antibodies overcome SARS-CoV-2 Omicron antigenic shift. Nature.

[41] Gray G., Collie S., Goga A., Garrett N., Champion J., Seocharan I., Bamford L., Moultrie H., Bekker L.-G. (2022). Effectiveness of Ad26.COV2.S and BNT162b2 Vaccines against Omicron Variant in South Africa. N. Engl. J. Med..

[42] El Sahly H.M., Baden L.R., Essink B., Doblecki-Lewis S., Martin J.M., Anderson E.J., Campbell T.B., Clark J., Jackson L.A., Fichtenbaum C.J. (2021). Efficacy of the mRNA-1273 SARS-CoV-2 Vaccine at Completion of Blinded Phase. N. Engl. J. Med..

[43] Baden L.R., El Sahly H.M., Essink B., Kotloff K., Frey S., Novak R., Diemert D., Spector S.A., Rouphael N., Creech C.B. (2020). Efficacy and Safety of the mRNA-1273 SARS-CoV-2 Vaccine. N. Engl. J. Med..

[44] Garcia-Beltran W.F., St Denis K.J., Hoelzemer A., Lam E.C., Nitido A.D., Sheehan M.L., Berrios C., Ofoman O., Chang C.C., Hauser B.M. (2022). mRNA-based COVID-19 vaccine boosters induce neutralizing immunity against SARS-CoV-2 Omicron variant. Cell.

[45] Tseng H.F., Ackerson B.K., Luo Y., Sy L.S., Talarico C.A., Tian Y., Bruxvoort K.J., Tubert J.E., Florea A., Ku J.H. (2022). Effectiveness of mRNA-1273 against SARS-CoV-2 Omicron and Delta variants. Nat. Med..

[46] Karbiener M., Farcet M.R., Zollner A., Masuda T., Mori M., Moschen A.R., Kreil T.R. (2022). Calibrated comparison of SARS-CoV-2 neutralizing antibody levels in response to protein-, mRNA-, and vector-based COVID-19 vaccines. NPJ Vaccines.

[47] Cerqueira-Silva T., Andrews J.R., Boaventura V.S., Ranzani O.T., de Araújo Oliveira V., Paixão E.S., Júnior J.B., Machado T.M., Hitchings M.D.T., Dorion M. (2022). Effectiveness of CoronaVac, ChAdOx1 nCoV-19, BNT162b2, and Ad26.COV2.S among individuals with previous SARS-CoV-2 infection in Brazil: A test-negative, case-control study. Lancet Infect. Dis..

[48] Mlcochova P., Kemp S.A., Dhar M.S., Papa G., Meng B., Ferreira I.A.T.M., Datir R., Collier D.A., Albecka A., Singh S. (2021). SARS-CoV-2 B.1.617.2 Delta variant replication and immune evasion. Nature.

[49] Bernal J.L., Andrews N., Gower C., Gallagher E., Simmons R., Thelwall S., Stowe J., Tessier E., Groves N., Dabrera G. (2021). Effectiveness of Covid-19 Vaccines against the B.1.617.2 (Delta) Variant. N. Engl. J. Med..

[50] UKHSA Gateway Number GOV-11226. https://assets.publishing.service.gov.uk/government/uploads/system/uploads/attachment_data/file/1050721/Vaccine-surveillance-report-week-4.pdf.

[51] Stephenson K.E., Le Gars M., Sadoff J., de Groot A.M., Heerwegh D., Truyers C., Atyeo C., Loos C., Chandrashekar A., McMahan K. (2021). Immunogenicity of the Ad26.COV2.S Vaccine for COVID-19. JAMA.

[52] Jongeneelen M., Kaszas K., Veldman D., Huizingh J., van der Vlugt R., Schouten T., Zuijdgeest D., Uil T., van Roey G., Guimera N. (2021). Ad26.COV2.S elicited neutralizing activity against Delta and other SARS-CoV-2 variants of concern. bioRxiv.

[53] Polinski J.M., Weckstein A.R., Batech M., Kabelac C., Kamath T., Harvey R., Jain S., Rassen J.A., Khan N., Schneeweiss S. (2022). Durability of the Single-Dose Ad26.COV2.S Vaccine in the Prevention of COVID-19 Infections and Hospitalizations in the US Before and During the Delta Variant Surge. JAMA Netw. Open.

[54] Echeverría G., Guevara Á., Coloma J., Ruiz A.M., Vasquez M.M., Tejera E., de Waard J.H. (2021). Pre-existing T-cell immunity to SARS-CoV-2 in unexposed healthy controls in Ecuador, as detected with a COVID-19 Interferon-Gamma Release Assay. Int. J. Infect. Dis..

[55] Weiskopf D., Katharina S.S., Matthijs P.R., Grifoni A., Nisreen M.A.O., Endeman H., van den Akker Johannes P.C., Molenkamp R., Marion P.G.K., van Gorp Eric C.M. (2020). Phenotype and kinetics of SARS-CoV-2–specific T cells in COVID-19 patients with acute respiratory distress syndrome. Sci. Immunol..

[56] Braun J., Loyal L., Frentsch M., Wendisch D., Georg P., Kurth F., Hippenstiel S., Dingeldey M., Kruse B., Fauchere F. (2020). SARS-CoV-2-reactive T cells in healthy donors and patients with COVID-19. Nature.

[57] Sette A., Crotty S. (2020). Pre-existing immunity to SARS-CoV-2: The knowns and unknowns. Nat. Rev. Immunol..

[58] Shafqat A., Shafqat S., Salameh S.A., Kashir J., Alkattan K., Yaqinuddin A. (2022). Mechanistic Insights Into the Immune Pathophysiology of COVID-19; An In-Depth Review. Front. Immunol..

[59] Mateus J., Grifoni A., Tarke A., Sidney J., Ramirez S.I., Dan J.M., Burger Z.C., Rawlings S.A., Smith D.M., Phillips E. (2020). Selective and cross-reactive SARS-CoV-2 T cell epitopes in unexposed humans. Science.

[60] Tan C.C.S., Owen C.J., Tham C.Y.L., Bertoletti A., van Dorp L., Balloux F. (2021). Pre-existing T cell-mediated cross-reactivity to SARS-CoV-2 cannot solely be explained by prior exposure to endemic human coronaviruses. Infect. Genet. Evol..

[61] Mahajan S., Kode V., Bhojak K., Karunakaran C., Lee K., Manoharan M., Ramesh A., Hv S., Srivastava A., Sathian R. (2021). Immunodominant T-cell epitopes from the SARS-CoV-2 spike antigen reveal robust pre-existing T-cell immunity in unexposed individuals. Sci. Rep..

[62] Dugas M., Grote-Westrick T., Vollenberg R., Lorentzen E., Brix T., Schmidt H., Tepasse P.-R., Kühn J. (2021). Less severe course of COVID-19 is associated with elevated levels of antibodies against seasonal human coronaviruses OC43 and HKU1 (HCoV OC43, HCoV HKU1). Int. J. Infect. Dis..

[63] Sagar M., Reifler K., Rossi M., Miller N.S., Sinha P., White L.F., Mizgerd J.P. (2021). Recent endemic coronavirus infection is associated with less-severe COVID-19. J. Clin. Investig..

[64] Loyal L., Braun J., Henze L., Kruse B., Dingeldey M., Reimer U., Kern F., Schwarz T., Mangold M., Unger C. (2021). Cross-reactive CD4^+^ T cells enhance SARS-CoV-2 immune responses upon infection and vaccination. Science.

[65] Vardhana S., Baldo L., William G.M., Wherry E.J. (2022). Understanding T-cell responses to COVID-19 is essential for informing public health strategies. Sci. Immunol..

[66] Altmann D.M., Boyton R.J. (2022). COVID-19 vaccination: The road ahead. Science.

[67] Kent S.J., Khoury D.S., Reynaldi A., Juno J.A., Wheatley A.K., Stadler E., John Wherry E., Triccas J., Sasson S.C., Cromer D. (2022). Disentangling the relative importance of T cell responses in COVID-19: Leading actors or supporting cast?. Nat. Rev. Immunol..

